# Determination, residue analysis and risk assessment of thiacloprid and spirotetramat in cowpeas under field conditions

**DOI:** 10.1038/s41598-022-07119-1

**Published:** 2022-03-02

**Authors:** Kailong Li, Wuying Chen, Wei Xiang, Tongqiang Chen, Min Zhang, Ying Ning, Yong Liu, Ang Chen

**Affiliations:** 1 Hunan Plant Protection Institute, Hunan Academy of Agricultural Science, Changsha, 410125 Hunan People’s Republic of China; 2 Crop Research Institute, Hunan Academy of Agricultural Science, Changsha, 410125 Hunan People’s Republic of China; 3 Hunan Testing Institute of Product and Commodity Supervision, Changsha, 410017 Hunan People’s Republic of China

**Keywords:** Environmental chemistry, Environmental sciences, Risk factors

## Abstract

The dissipation and residue levels of thiacloprid, spirotetramat and its four metabolites residues in cowpeas were investigated under field conditions. The QuEChERS technique with high-performance liquid chromatography tandem mass spectrometry (HPLC–MS/MS) was used to detect thiacloprid, spirotetramat and its four metabolites residues content in cowpeas. The recoveries were 81.3–95.1% at a spike level of 0.005–0.5 mg/kg, the relative standard deviations (RSDs) were 2.1–9.5%. The dissipation kinetics data showed that thiacloprid and spirotetramat in cowpeas were degraded with the half-lives of 1.14–1.54 days and 1.25–2.79 days. The terminal residues of thiacloprid and spirotetramat were 0.0255–0.4570 mg kg^−1^ and 0.0314–0.3070 mg kg^−1^ after application 2 times with a pre-harvest interval (PHI) of 3 days under the designed dosages. The chronic and acute dietary exposure assessment risk quotient (RQ) values of thiacloprid in cowpeas for different consumers were 2.44–4.41% and 8.72–15.78%, respectively, and those of spirotetramat were 1.03–1.87% and 0.18–0.32%, respectively, all of the RQ values were lower than 100%. The dietary risk of thiacloprid through cowpeas to consumers was higher than spirotetramat. The results from this study are important reference for Chinese governments to develop criteria for the safe and rational use of thiacloprid and spirotetramat, setting maximum residue levels (MRLs), monitoring the quality safety of agricultural products and protecting consumer health.

## Introduction

Pesticides are widely used to ensure high crop yields, but at the same time they also bring a serious safety risk to the human health and environment. In general, the pesticide residues exposure through the food diet is much higher than other exposure routes, such as air and drinking water, and vegetables were one of the main sources of food dietary exposure to pesticide residues^1,2^. Therefore, it is necessary to carry out the assessment of the residue level and dissipation behavior of pesticides after applying to vegetables, and establish the corresponding maximum residue limit (MRL) through these basic data. The establishment of MRL will provide an important technical guarantee for strengthening agricultural product quality and safety supervision and maintaining the sustainable development of agricultural international trade in China^3,4^.

Cowpeas (*Vigna unguiculate* [L.] Walp.) are very popular vegetables that are rich in protein and carbohydrate, together with an amino acid pattern complementary to that of cereal grains, are important nutritional component in the human diet^5,6^. The high temperature climate characteristics of the cowpeas planting season make cowpeas pests serious, so a large number of chemical pesticides are applied to control the pests on the cowpeas, such as aphids, thrips, and liriomyza. Several new systemic insecticides, including thiacloprid and spirotetramat, have been proved to have good control effects on sucking pests^7,8^. And these pesticides will accumulate in agricultural products, causing potential ecological and food safety risks.

Thiacloprid, (Z)-3-(6-chloro-3-pyridylmethyl)-1,3-thiazolidin-2-ylidenecyanamide (IUPAC), the first chloronicotinyl insecticide, is a nicotinic acetylcholine receptor agonist in the central nervous system, thus disturbing synaptic signal transmission. It can not only control sucking pests, such as aphids and whiteflies, but also weevils, leafminers and various species of beetles, and is numerously used in China^9,10^.

Spirotetramat, cis-3-(2,5-dimethlyphenyl)-8-methoxy-2-oxo-1-azaspiro [4.5] dec-3-en-4-yl- ethyl carbonate (BYI08330) (IUPAC), is a tetramic acid insecticide, that has a two-way systemic conductivity property, providing complete systemic protection from a broad spectrum of sucking pests^8^. Spirotetramat-enol (B-enol), spirotetramat-mono-hydroxy (B-mono), and spirotetramat-keto-hydroxy (B-keto) and spirotetramat-enol-glucoside (B-glu) are the main metabolites of spirotetramat in plants^11^.

Monitoring residue levels in vegetables is very important mainly because of the toxicity of the pesticide itself or its metabolites. The JMPR (Joint FAO/WHO Meeting on Pesticide Residues) report shows that thiacloprid is an acute contact and stomach poison, was moderate acute toxicity to rats after oral (LD_50_, 396–836 mg kg^−1^ b.w.)^12^. The maternal rat toxicity of spirotetramat was ≥ 40 mg kg^−1^ b.w. day^−1^, and the metabolite B-enol was likely caused male rat reproductive toxicity^13^. Consequently, studying the residue degradation dynamics of thiacloprid and spirotetramat on cowpeas is helpful to guide the scientific pesticide use on cowpea and make a reasonable assessment of the safety of their residues.

Based on the JMPR reports, the residue definition of spirotetramat and thiacloprid for plant commodities are spirotetramat plus its all metabolites and thiacloprid, respectively^12,13^. The dissipation behavior of thiacloprid has been investigated in cabbage, tomato, Asian pear, tea and citrus^14–18^. The dissipation behavior of spirotetramat has been investigated in mango, chilli, grape, pistachio and citrus^19–23^. However, the four metabolites of spirotetramat were ignored in these vegetable and fruit dissipation studies. Thiacloprid and spirotetramat are regularly used in cowpeas, while there are still few studies on the evaluation and monitoring of these two compounds in cowpeas, and the MRL of thiacloprid in cowpeas has not yet been established in Codex Alimentarius Commission (CAC) and China. The European Union (EU) MRL of thiacloprid in Beans (with pods) is 0.4 mg kg^−1^, the MRL of spirotetramat in legume vegetables is 1.5 mg kg^−1^ (EU, CAC and Korea), 2.0 mg kg^−1^ (Australian), 3.0 mg kg^−1^ (Japan) and the MRL of spirotetramat in cowpeas is 5.0 mg kg^−1^ (China, a temporary limit value).

Thiacloprid and spirotetramat residues could be extracted from different vegetables and fruits matrices by acetonitrile. The commonly used analytical methods for thiacloprid and spirotetramat are liquid chromatography (LC)^15,20^ and LC-mass spectrometry (LC–MS/MS)^11,22–24^. In addition, we can see that the improved QuEChERS methods have been successfully used to recover thiacloprid or spirotetramat from many vegetables and fruits, such as cabbage^14^, apple^25^, tomato^26^, grape^21^, citrus^18^, cucumber^26^ and green onion^27^. However, the method for simultaneously determining thiacloprid, spirotetramat and its four metabolites in vegetables was not established.

In this study, we aim to establish a method for detecting thiacloprid, spirotetramat and its four metabolites residues in cowpeas and investigated the degradation dynamics and final residues on cowpeas from different crop-growing environment (open-field and greenhouse) in China. Additionally, we would conduct a short-term and long-term dietary risk assessment for different genders and ages consumers in China and develop MRLs criteria of thiacloprid and spirotetramat in cowpeas to provide a scientific basis to minimize health risks to consumers.

## Materials and methods

### Materials and reagents

**Scheme 1:**
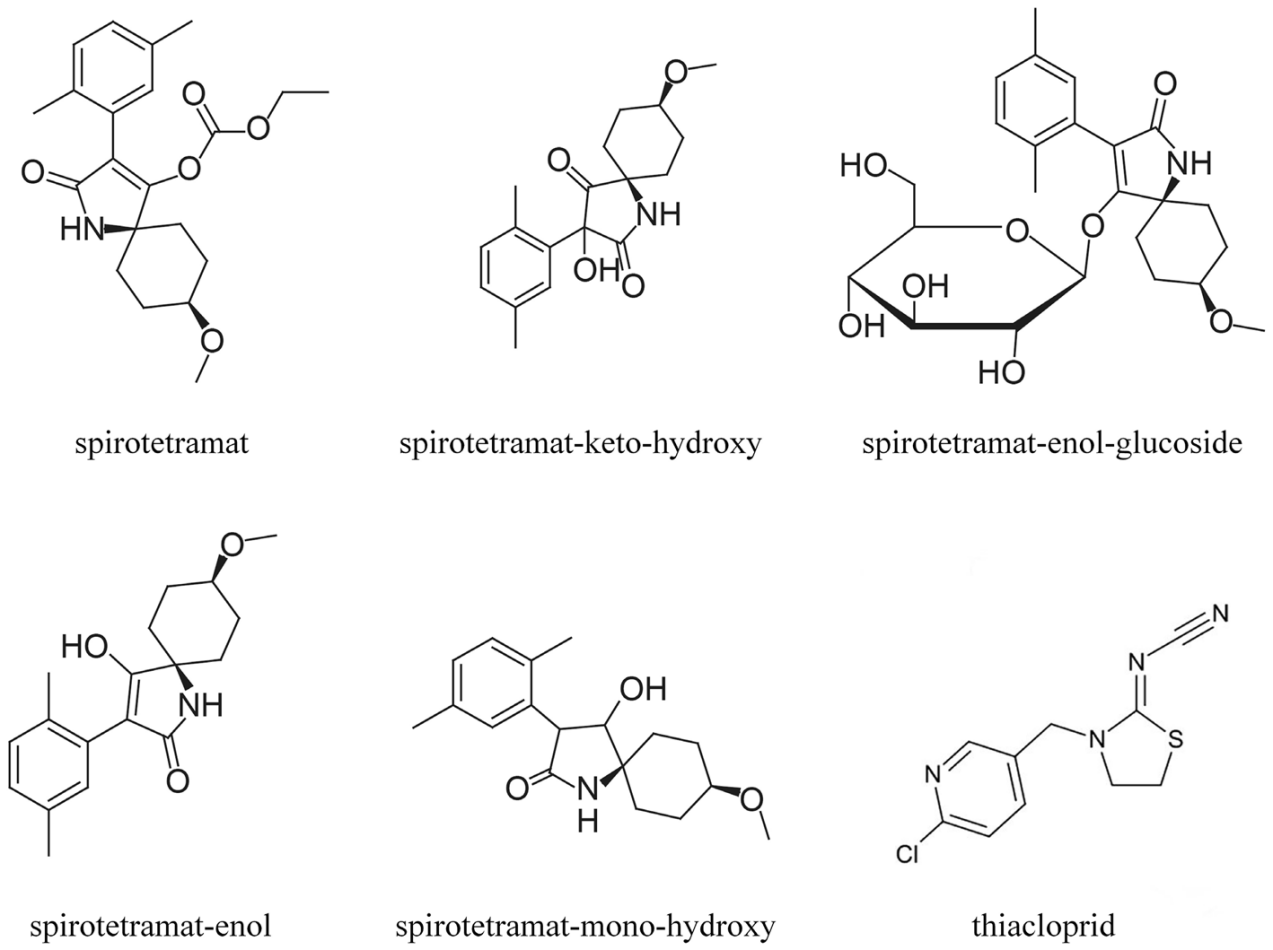
Structural formula of thiacloprid, spirotetramat and its four metabolites.

The suspensions concentrate (SC) formulations of thiacloprid and spirotetramat (22% SC) for field trials were provided by Anhui Fengle Agrochemical Co., Ltd. (Anhui, China). The active ingredient 1 spirotetramat content is 11%, and the active ingredient 2 thiacloprid content is 11%. Spirotetramat (99.2% purity), B-enol (99.8% purity), B-keto (92.8% purity), B-mono (98.2% purity) and B-glu (89.6% purity) standard material were purchased from Dr. Ehrenstorfer (Augsburg, Germany), thiacloprid (99.0% purity) standard material was purchased from ChemService (West Chester, USA). The HPLC grade acetonitrile and methanol were purchased from Merck (Darmstadt, Germany). Anhydrous magnesium sulphate (MgSO_4_), sodium chloride (NaCl) and formic acid were of analytical grade and purchased from Sinopharm Chemical Reagent (Beijing, China). Primary secondary amine (PSA), Octadecylsilane (C18) and Graphitized Carbon (GCB) sorbent were purchased from Agela Technologies (Tianjin, China). Stock standard solutions (100.0 mg L^−1^) of thiacloprid, BYI08330, B-enol, B-keto, B-mono and B-glu were dissolved in acetonitrile and stored at − 20 °C. The chemical formulas of thiacloprid, spirotetramat and its four metabolites are shown in Scheme 1.

### Field trial

To investigate the residue levels of thiacloprid and spirotetramat in cowpeas, field trials were carried out during June to August 2019 at different locations under good agricultural practice (GAP). The representative cowpea-growing provinces are Hunan (open-field environment, 113°10′ E and 28° 9′ N), Guizhou (open-field environment, 106° 62′ E and 26° 31′ N), Shandong (greenhouse environment, 116° 42′ E and 36° 46′ N), Zhejiang (greenhouse environment, 119° 85′ E and 30° 25′ N), Liaoning (greenhouse environment, 123° 17′ E and 43° 3′ N), Hebei (greenhouse environment, 114° 78′ E and 37° 35′ N), Henan (greenhouse environment, 113° 63′ E and 34° 95′ N), and Jiangsu (open-field environment, 119° 14′ E and 32° 17′ N). The field trials were designed according to the Guideline on Pesticide Residue Trials in China (NY/T 788-2018)^28^. Each treatment had three replicate plots and one control plot with 30 m^2^ area per plot. A buffer zone (30 m^2^) was utilized to separate the plots to avoid cross-contamination.

In the degradation experiments, cowpeas were sprayed with a 22% SC of thiacloprid and spirotetramat at a dosage of 144 g a.i. ha^−1^ in Hunan, Guizhou, Zhejiang and Shandong. Then, 2 kg of representative cowpeas samples were collected randomly at 2 h and 1, 3, 5, 7 and 14 days after treatment. In the terminal residue experiments, cowpeas were sprayed with 22% SC of thiacloprid and spirotetramat at a dosage of 144 g a.i. ha^−1^ (recommended high dose) for 2 times, and the application interval is 7 days. The recommended application preharvest interval was 3 days. Representative cowpeas were collected at 3, 5 days after the last spraying. Collected cowpeas samples were transported to the laboratory within 8 h, chopped and maintained at − 20 °C until further analysis. We have the permissions to collect and manage all the samples of cowpeas samples. Among them, the samples from Hunan were collected by us and the samples from other provinces were collected by entrusting cooperative institute.

### Sample extraction by QuEChERS pretreatment

A 10 g subsample of the cowpea samples was put into a 100 mL PTFE centrifuge tube. Next, 25 mL acetonitrile were added, and the mixture was homogenized with a high-speed dispersing machine for 3 min at 1200 strokes min^−1^. Subsequently, 4 g of anhydrous MgSO_4_ and 1 g of NaCl were added and vortexed with a multi-tube vortex mixer for 1 min. After centrifuging the tubes at 4000×*g* for 5 min, a 1.0 mL supernatant was transferred to a new centrifuge tube and diluted with 1 mL acetonitrile. An aliquot of the supernatant (1 mL) was transferred to a single-use 2.0 mL PTFE centrifuge tube containing 50 mg PSA sorbents and 150 mg anhydrous MgSO_4_. The vortex step was operated with vortex mixer for 1 min and centrifuged at 10,000×*g* for 5 min. After that, the upper layer of the prepared sample was filtered through a 0.22 μm nylon syringe filters for LC–MS/MS analysis.

In this study, the extraction efficiency was compared between ethyl acetate, acetonitrile and acetonitrile-ethyl acetate (1:1). The experiment investigated the adsorption effects of commonly used adsorbents (50 mg PSA + 150 mg anhydrous MgSO_4_, 50 mg C18 + 150 mg anhydrous MgSO_4_, 50 mg PSA + 10 mg GCB + 150 mg anhydrous MgSO4 and 50 mg C18 + 10 mg GCB + 150 mg anhydrous MgSO_4_) for thiacloprid, spirotetramat and four metabolites of QuEChERS pretreatment methods. The resolution and response strength of four mobile phase systems (methanol–water, methanol-0.1% formic acid aqueous solution, acetonitrile–water, and acetonitrile-0.1% formic acid aqueous solution) to target compounds were investigated.

### Instrumentation

The analyses were conducted on an AB Sciex 4500Q Trap LC–MS/MS system (Framingham, USA) equipped with an electrospray ionization (ESI^+^) source. The ZORBAX RRHD Eclipse plus C18 column (3.0 × 100 mm id, 1.8 μm particle size; Agilent Technologies, Santa Clara, CA, USA) was utilized under the temperature of 40 °C. The mobile phase consisted of acetonitrile (A) and 0.1% (v/v) formic acid aqueous solution (B) at a flow rate of 0.4 min L^−1^ with an injection volume of 10 μL. Elution was performed in the gradient mode (0–0.9 min, 30% A; 0.9–1.0 min, 90% A; 1.0–3.1 min, held at 90% A; 3.1–3.2 min, 30% A3.2–5.0 min, held at 30% A), total analysis time was 5.0 min. The analysis was conducted with electrospray ionization (ESI) in positive ion mode. All compounds were detected under multiple reactions monitoring (MRM) mode. Typical instrumentation conditions: ion spray voltage 5500 V, curtain gas 45 psi, ion source gas 1 and gas 2 at 40 psi and ion source temperature 550 °C.

### Method validation

Validation characteristics were evaluated by evaluating specificity, linearity, limit of detection (LOD), limit of quantification (LOQ), matrix affect (ME), accuracy and precision. The standard solutions were dissolved in acetonitrile as solvent standard solutions and the matrix-matched standard solutions were prepared by dissolving the standard solutions in control matrix (cowpeas) extract solutions. The concentrations of 0.0005–0.5 mg L^−1^ of the solvent standard solution and the matrix-matched standard solutions were each injected three times.

Recovery experiment was conducted for evaluating accuracy and precision of method, analyzing spiked samples at three spiked concentration levels (0.005 mg kg^−1^, 0.05 mg kg^−1^ and 0.5 mg kg^−1^) with five replications. The spiked sample was obtained by adding the appropriate volume of the mixed standard solution of thiacloprid, BYI08330 and its four metabolites to homogenized sample before extraction. The precision of the developed method was evaluated by measuring the relative standard deviations (RSDs).

### Statistical analysis

The standard curve slope (S) of matrix-matched standard solution with the solvent standard solution was calculated according the following equation to evaluate the matrix effect (ME)^29,30^.

1 $${\text{ME }}\left( \% \right) \, = \, \left( {{\text{S}}_{{\text{in matrix}}} - {\text{S}}_{{\text{in solvent}}} } \right)/{\text{S}}_{{\text{in solvent}}} \times { 1}00\% .$$

The dissipation dynamic of thiacloprid and spirotetramat in cowpeas was evaluated by using the first-order kinetics regression analysis^31^:

2 $${\text{C}}_{{\text{T}}} = {\text{ C}}_{0} \times {\text{ e}}^{{ - {\text{kT}}}} ,$$

3 $${\text{T}}_{{{1}/{2}}} = {\text{ ln2}}/{\text{k}}.$$

C_0_ (mg kg^−1^) is the initial concentration and k (day^−1^) is the pesticide dissipation rate constant, C_T_ (mg kg^−1^) is the residual concentration at time point T (day) and T_1/2_ is the pesticide half-life of pesticide degradation.

### Dietary intake risk assessment

In the risk assessment of chronic dietary exposure, the NEDI was calculated based on the STMR using Eqs. (4) and (5).

4 $${\text{NEDI }} = \, \left( {\Sigma {\text{STMRi }} \times {\text{ Fi}}} \right)/{\text{b}}.{\text{w}}.,$$

5 $${\text{RQ}}_{{\text{c}}} = {\text{ NEDI}}/{\text{ADI}}.$$

In the risk assessment of acute dietary exposure, the NESTI was calculated based on the HR using Eqs. (6) and (7).

6 $${\text{NESTI }} = {\text{ HR }} \times {\text{ Fi}}/{\text{b}}.{\text{w}}.,$$

7 $${\text{RQ}}_{{\text{a}}} = {\text{ NESTI}}/{\text{ARfD}}.$$

Here, RQ is the risk quotient, STMRi (mg kg^−1^) is median residue level of supervised trials, HR (mg kg^−1^) is the highest residue of supervised trials, Fi (kg) is the consumption of one crop for the different age groups population, NEDI (mg kg^−1^ b.w.) is the national estimated daily intake, ADI (mg kg^−1^ b.w.) is the acceptable daily intake, ARfD (mg kg^−1^ b.w.) is acute reference dose, b.w. (kg) is the average body weight and NESTI (mg kg^−1^ b.w.) is the national estimated short term intake.

## Results and discussion

### Optimization of sample extraction, purification and LC conditions

The extraction efficiency results showed that when acetonitrile was used as the extraction solvent, the extraction rate of the six target pesticides was 81.7–102.3%, when ethyl acetate and acetonitrile-ethyl acetate was used as the extraction solvent, the recovery rate of B-enol were only 43% and 23.3%. This may be due to the fact that ethyl acetate is insoluble in water, resulting in the inability to completely extract the strongly polar compound B-enol from the matrix. Therefore, acetonitrile was finally selected as the extraction solvent.

The recovery results showed that the recovery rate of C18 adsorbent to B-enol and B-glu was low (65. 6–81.8%), PSA + GCB combination has a low recovery rate of B-glu (63.2–74.4%), the recovery rate of C18 + GCB combination to B-enol and B-glu was extremely low (45.1–64.3%), the recovery rate of PSA adsorbent to 6 compounds are all above 80%. Therefore, in this study, PSA adsorbent + 150 mg anhydrous MgSO4 was selected to obtain good adsorption effect and recovery rate of thiacloprid, spirotetramat and its four metabolites.

**Figure 1:**
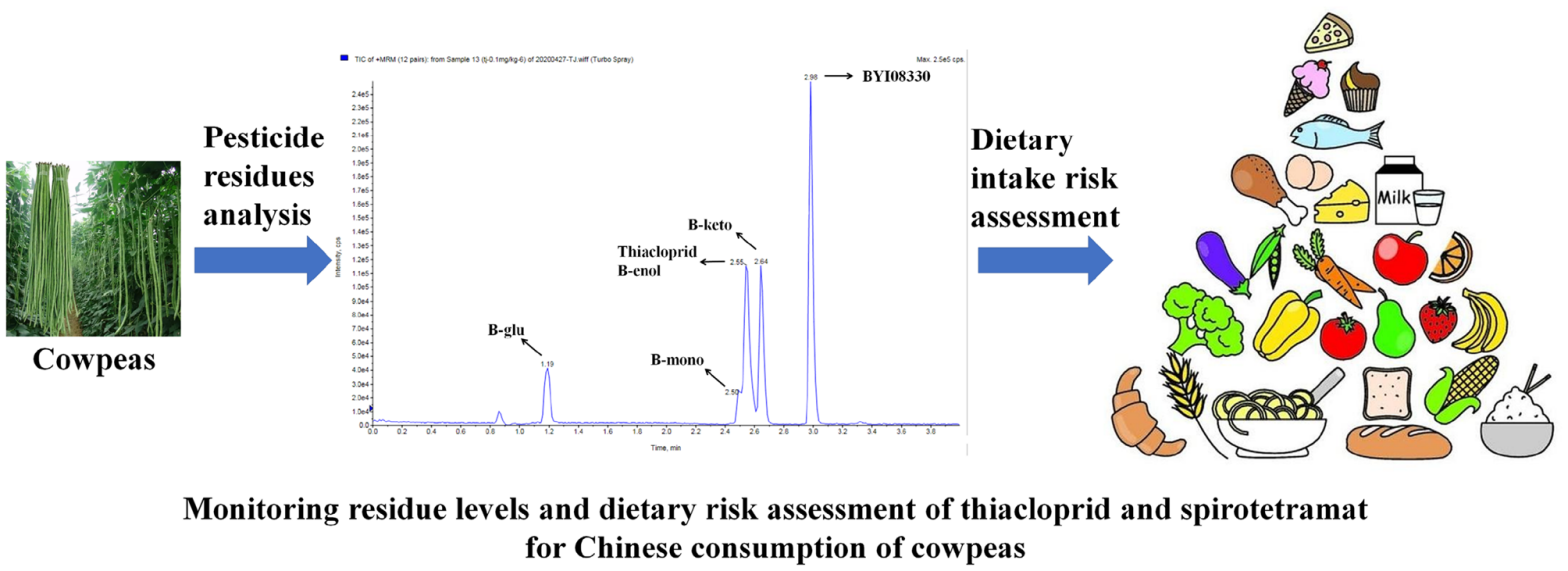
Sketch map.

The mobile phase systems optimization results show that when methanol is used as the organic phase, the peak shape of B-glu is asymmetric and the peak is broadened, which affects the quantitative results. When acetonitrile is used as the organic phase, adding 0.1% formic acid to the water phase can promote the formation of [M + H]^+^ ion peaks and improves the sensitivity of target pesticide detection. Therefore, the mobile phase uses acetonitrile-0.1% formic acid aqueous solution in a single run of 5.0 min, with retention time of 2.54 (thiacloprid), 2.98 min (BYI08330), 2.54 min (B-enol), 2.63 min (B-keto), 2.48 min (B-mono) and 1.18 min (B-glu), as shown in Fig. 1 and Table 1.

**Table 1:** Optimized parameters for MS/MS of thiacloprid, spirotetramat and its metabolites.

Analyte	Precursor ion, m/z	Product ion (CE), m/z		Declustering potential (V)	Retention time (Min)
		Quantitation	Confirmation		
Thiacloprid	253.0	125.9 (27)	89.9 (53)	71	2.55
BYI08330	374.1	302.1 (21)	330.1 (21)	91	2.98
B-mono	304.1	254.1 (23)	91.0 (67)	96	2.50
B-enol	302.1	216.0 (35)	270.0 (29)	121	2.55
B-keto	318.1	300.0 (15)	214.1 (39)	66	2.64
B-glu	464.1	302.1 (17)	215.9 (57)	31	1.19

*CE* collision energy (eV).

### Optimization of MS/MS parameters

The ionization effect of the positive and negative ion scanning mode on the 6 target compounds were investigated and result showed that the target compound had a higher response in the positive ion mode. In order to improve the ionization efficiency of the target compound, the source parameters were optimized. In the positive ion mode, the compound is fully scanned by needle pump injection, and the stable [M + H]^+^ molecular ion was obtained through the MS scan. After the parent ion was determined, the declustering potential (DP) in the MRM mode was optimized. In the secondary mass spectrometry, fragmentation reactions such as fragmentation or rearrangement of the precursor ion occur, resulting in different m/z ion fragments. The two fragment ions with the highest response and the least interference were selected as the qualitative and quantitative ions and the collision energy (CE) corresponding to each ion was optimized. Table 1 shows the optimized parameters for MS/MS of thiacloprid, spirotetramat and its metabolites.

### Method validation

The linearity of the calibration curves of thiacloprid, BYI08330 and its four metabolites were good within the range of 0.0005–0.5 mg L^−1^. The correlation coefficients (R^2^) of solvent and matrix-matched standards were all ≥ 0.99 and the calibration curves were showed in Fig. S1. The cowpeas matrix effect for thiacloprid, BYI08330, B-enol, B-keto, B-mono and B-glu is − 36.9%, − 43.7%, − 47.3%, − 26.2%, − 23.2% and − 54.5%, respectively (Table 2). The ME value of thiacloprid, BYI08330, B-enol, B-keto, B-mono and B-glu are both lower than − 20.0%, which indicated that the matrix effect is signal suppression^32^. Therefore, matrix-matched standard calibrations were prepared to eliminate influence of matrix on the identification and quantification of thiacloprid, BYI08330 and its four metabolites in cowpeas.

**Table 2:** Regression parameters for thiacloprid and spirotetramat calibration curve.

Analyte	Matrix	Regression equation	R^2^	Calibration range (mg L^−1^)	Matrix effect (%)
Thiacloprid	Acetonitrile	Y = 11,494,165.2327X + 100,035.4580	0.9961	0.0005–0.5	− 36.9
	Cowpeas	Y = 7,247,506.2444X + 38,335.8697	0.9987	0.0005–0.5	
BYI08330	Acetonitrile	Y = 10,603,392.5111X + 52,610.7940	0.9987	0.0005–0.5	− 43.7
	Cowpeas	Y = 7,379,105.0854X + 32,613.5420	0.9993	0.0005–0.5	
B-mono	Acetonitrile	Y = 3,606,228.9092X + 9240.8236	0.9996	0.0005–0.5	− 23.2
	Cowpeas	Y = 2,770,547.1943X + 5435.0421	0.9998	0.0005–0.5	
B-enol	Acetonitrile	Y = 8,819,902.7861X + 37,326.4942	0.9990	0.0005–0.5	− 47.3
	Cowpeas	Y = 4,647,123.1349X + 5032.5853	0.9999	0.0005–0.5	
B-keto	Acetonitrile	Y = 13,356,980.5043X + 14,540.4991	1.000	0.0005–0.5	− 26.2
	Cowpeas	Y = 9,853,614.1088X + 14,104.5519	0.9999	0.0005–0.5	
B-glu	Acetonitrile	Y = 6,110,554.9822X − 26,772.3184	0.9967	0.0005–0.5	− 54.5
	Cowpeas	Y = 2,781,697.8952X + 2031.1933	0.9999	0.0005–0.5	

At different spike levels (0.005–0.5 mg kg^−1^), the recovery of thiacloprid, BYI08330, B-mono, B-enol, B-keto, and B-glu were 81.3–92.4%, 82.4–94.4%, 86.4–93.4%, 85.4–92.3%, 89.6–95.1% and 88.0–91.6%, respectively. The corresponding RSDs were 2.1–5.7%, 2.3–9.5%, 1.8–9.4%, 1.3–7.4%, 1.1–6.2% and 4.3–7.6%, respectively (Table 3). According to NY/T 788-2018^28^, LOD definition was the lowest standard curve concentration, and LOQ definition was the lowest spike level. These results showed the LOD of thiacloprid, BYI08330 and its four metabolites was 0.0005 mg L^−1^, and the LOQ were 0.005 mg kg^−1^. These results demonstrated that this method could satisfy the requirements of the Guideline on Pesticide Residue Trials (NY/T 788-2018)^28^ in China, was suitable for residue analysis of thiacloprid, BYI08330 and its four metabolites in cowpeas.

**Table 3:** Recovery, RSD and LOQ of thiacloprid and spirotetramat in cowpeas (n = 5).

Analyte	Spiked level (mg kg^−1^)	Average recovery (%)	RSD (%)	LOD (mg L^−1^)	LOQ (mg kg^−1^)
Thiacloprid	0.005	81.3	2.1	0.0005	0.005
	0.05	85.6	2.4		
	0.5	92.4	5.7		
BYI08330	0.005	87.6	9.5	0.0005	0.005
	0.05	82.4	4.4		
	0.5	94.4	2.3		
B-mono	0.005	93.4	9.4	0.0005	0.005
	0.05	86.4	2.8		
	0.5	90.6	1.8		
B-enol	0.005	86.1	5.7	0.0005	0.005
	0.05	85.4	1.3		
	0.5	92.3	7.4		
B-kate	0.005	89.6	6.2	0.0005	0.005
	0.05	90.7	1.1		
	0.5	95.1	4.6		
B-glu	0.005	88.0	7.6	0.0005	0.005
	0.05	89.9	4.3		
	0.5	91.6	4.8		

### Dissipation kinetics of thiacloprid and spirotetramat in cowpeas

**Figure 2:**
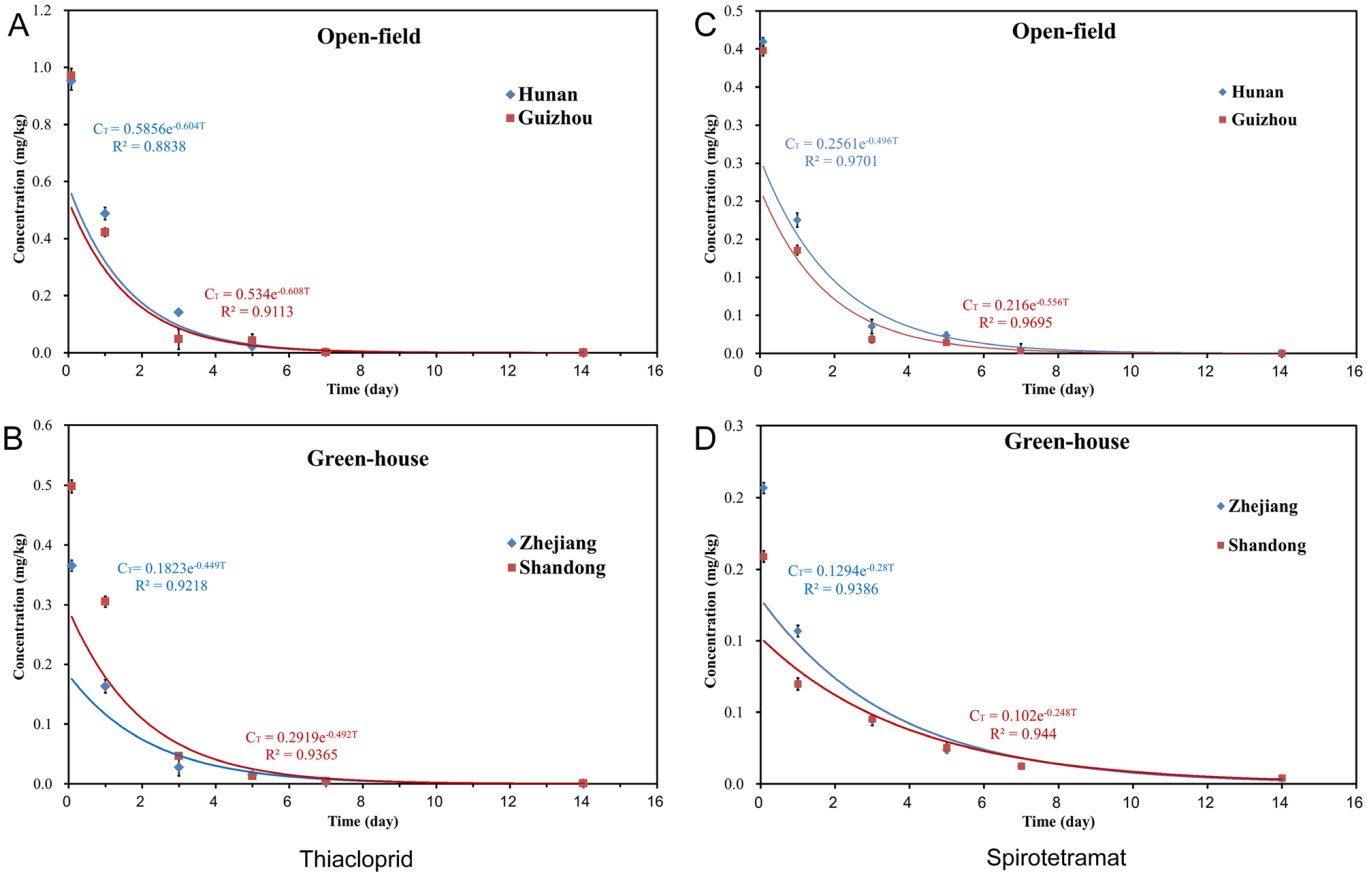
Dissipation kinetic curves of thiacloprid and spirotetramat in cowpeas.

Thiacloprid and spirotetramat SC (22%) was applied to the cowpeas in four regions (Guizhou, Hunan, Zhejiang and Shandong) at a concentration that was the recommended highest dose, and samples were collected at different time intervals for analysis. The related parameters are listed in Table 4. The initial deposited amounts of thiacloprid in cowpeas were 0.9702 mg kg^−1^ (Guizhou), 0.9519 mg kg^−1^ (Hunan), 0.3654 mg kg^−1^ (Zhejiang) and 0.4978 mg kg^−1^ (Shandong), and the residual content gradually decreased over time. The initial deposited amounts of BYI08330 in cowpeas were 0.398 mg kg^−1^ (Guizhou), 0.4095 mg kg^−1^ (Hunan), 0.2068 mg kg^−1^ (Zhejiang), 0.1589 mg kg^−1^ (Shandong), and the residual content gradually decreased over time. The degradation rates of thiacloprid ranged from 95.6 to 97.7% on 5 days after application and were lower than their LOQs on 7 days after application. The degradation rates of the parent compound BYI08330 ranged from 84.0 to 96.7% on 5 days after application and were lower than their LOQs on 10 days after application. The degradation process of thiacloprid and BYI08330 were consistent with the first-order kinetics equation, and Fig. 2 shows that a fast initial decrease in both pesticides was followed by a slower decline. The correlation coefficients of thiacloprid and BYI08330 among cowpeas in four regions were ≥ 0.8838 and ≥ 0.9386, and the corresponding half-life values were 1.14–1.54 days and 1.25–2.79 days, respectively. The dissipation dynamics of thiacloprid and BYI08330 in other crops also showed a similar pattern. The BYI08330 half-lives in pepper^11^ and herbs^24^ were 1.21 days and 0.51–0.83 days. The thiacloprid half-lives in cabbage^14^, tomato^15^, eggplant^33^ and pepper^11^ were 1.3–1.6 days, 0.83–1.79 days, 0.47–0.50 days and 0.81 days, respectively. The shortest half-life (1.14 days) and the longest half-life (1.54 days) of thiacloprid occurred for cowpea samples from Guizhou (open-field) and Zhejiang (greenhouse), respectively. The shortest half-life (1.25 days) and the longest half-life (2.79 days) of BYI08330 occurred for the cowpea samples from Guizhou (open-field) and Zhejiang (greenhouse), respectively. The initial deposited amounts of thiacloprid and BYI08330 in the open-field (Guizhou and Hunan) were all higher than those in the greenhouse (Zhejiang and Shandong). The degradation ratio of thiacloprid and spirotetramat in the open-field in in Guizhou and Hunan was higher than that in the greenhouse in Zhejiang and Shandong. There were differences in the half-life and degradation ratio of thiacloprid and spirotetramat between open-field and greenhouse growth environment. This finding may be related to temperature, humidity, application time, light, microorganisms, and other factors in different environments^34,35^.

**Table 4:** Dissipation kinetics of thiacloprid and spirotetramat in cowpeas.

Analyte	Environment	Location	Equation	Coefficient (R^2^)	Half-life (T_1/2_)
Thiacloprid	Open-field	Guizhou	C_T_ = 0.5340e^−0.608T^	0.9113	1.14
		Hunan	C_T_ = 0.5856e^−0.604T^	0.8838	1.15
	Greenhouse	Zhejiang	C_T_ = 0.1823e^−0.449T^	0.9218	1.54
		Shandong	C_T_ = 0.2919e^−0.492T^	0.9365	1.41
BYI08330	Open-field	Guizhou	C_T_ = 0.2160e^−0.556T^	0.9695	1.25
		Hunan	C_T_ = 0.2561e^−0.496T^	0.9701	1.40
	Greenhouse	Zhejiang	C_T_ = 0.1294e^−0.280T^	0.9386	2.48
		Shandong	C_T_ = 0.1020e^−0.248T^	0.9440	2.79

**Figure 3:**
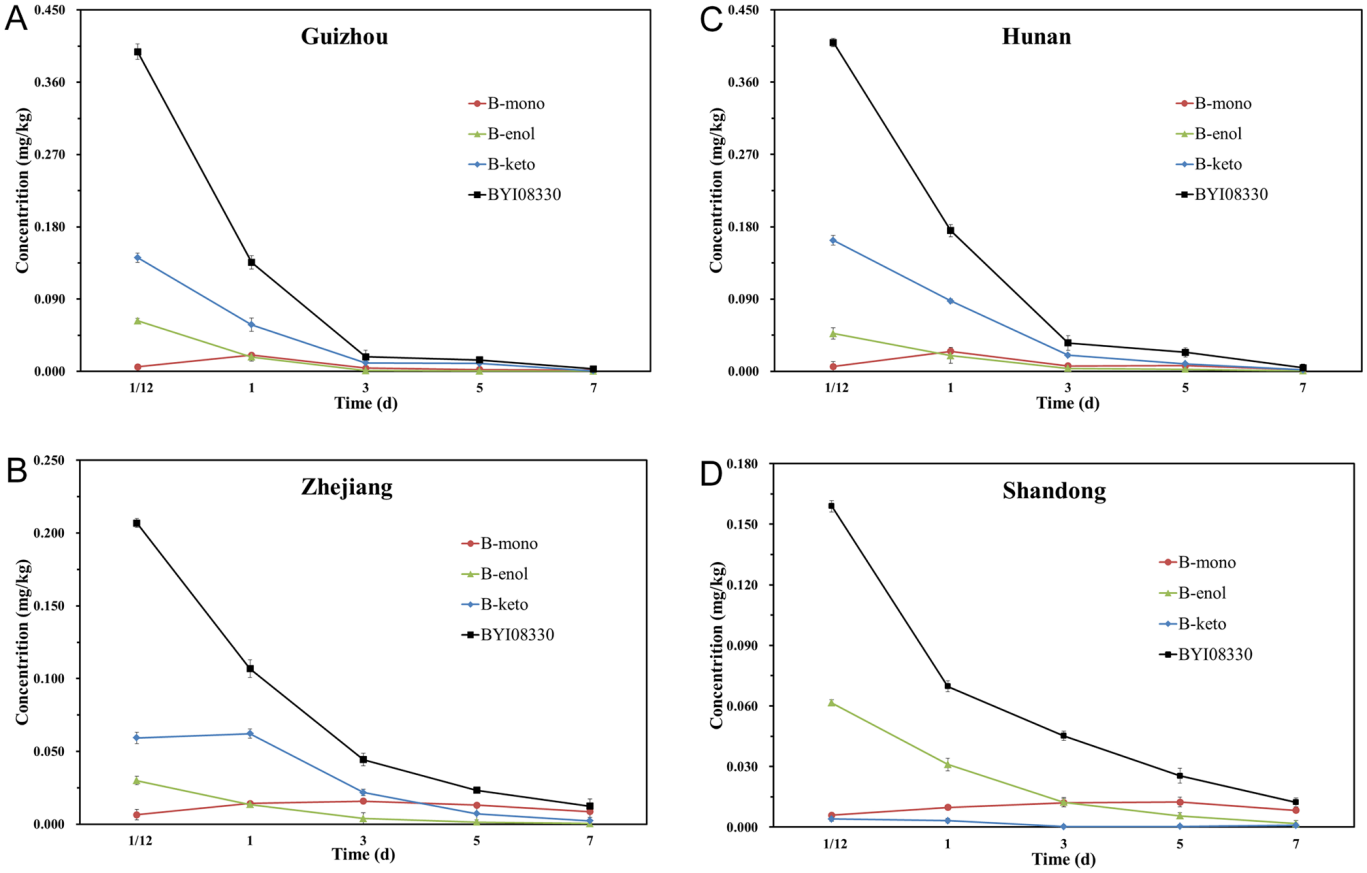
Dissipation of spirotetramat and its metabolites in cowpeas.

In this field experiment, BYI08330’s four metabolites were also detected in cowpea samples in different time and locations. The previous studies showed that the cleavage of the ester group in BYI08330 yields B-enol, hydroxylation of the tetramic acid moiety resulted in B-keto and B-mono, B-enol conjugated to glucose yielded B-glu^11^. In the dissipation cowpea samples, the detected B-glu concentration was below the LOD, B-mono increased first and then decreased continuously throughout the entire dissipation period, whereas B-enol and B-keto were decreased continuously throughout the entire period (Fig. 3).

### Final residue distributions of thiacloprid and spirotetramat in cowpeas

The terminal residues of thiacloprid and BYI08330 (sum of BYI08330, B-enol, B-keto, B-mono and B-glu, expressed as BYI08330) were positive after applying the 22% SC of thiacloprid and spirotetramat at the recommended dosage 144 g a.i. ha^−1^. The terminal residue level of thiacloprid and BYI08330 were listed in Table 5. The data showed that the terminal residue of thiacloprid in cowpeas were 0.0255–0.4570 mg kg^−1^ and 0.0007–0.2058 mg kg^−1^ at PHI 3 days and 5 days, respectively. And the residual amounts of BYI08330 (sum of BYI08330, B-enol, B-mono, B-keto and B-glu) in cowpeas were 0.0314–0.3070 mg kg^−1^ and 0.0334–0.1407 mg kg^−1^ at PHI 3 days and 5 days, respectively. The STMRs of thiacloprid and BYI08330 in cowpeas at PHI 3 days were 0.0426 mg kg^−1^ and 0.0903 mg kg^−1^, and the highest residues (HRs) were 0.4570 mg kg^−1^ and 0.3070 mg kg^−1^. The STMRs of thiacloprid and BYI08330 in cowpeas at PHI 5 days were 0.0267 mg kg^−1^ and 0.0.0702 mg kg^−1^, and the highest residues (HRs) were 0.2058 mg kg^−1^ and 0.1407 mg kg^−1^. The STMRs and HRs values of thiacloprid in cowpeas were all below the EU maximum residue level (0.4 mg kg^−1^)^36^, and the STMRs and HRs values of spirotetramat in cowpeas were below the CAC (1.5 mg kg^−1^)^37^, EU (1.5 mg kg^−1^)^36^, Korea (1.5 mg kg^−1^)^38^, Australian (2.0 mg kg^−1^)^39^, Japan (3.0 mg kg^−1^)^40^ and China (5.0 mg kg^−1^)^41^ maximum residue level.

**Table 5:** Final residues of thiacloprid and spirotetramat in cowpeas.

Analyte	Dosage g a.i ha^−1^	Times	PHI	Residues data in eight region* (n = 3) (mean value, mg kg^−1^)	STMR (mg kg^−1^)	HR (mg kg^−1^)
Thiacloprid	144	2	3	0.0255, 0.0287, 0.0319, 0.0371	0.0426	0.4570
				0.0481, 0.1679, 0.1852, 0.4570		
			5	0.0007, 0.0013, 0.0018, 0.0100	0.0267	0.2058
				0.0434, 0.0570, 0.1961, 0.2058		
BYI08330^#^	144	2	3	0.0314, 0.0409, 0.0655, 0.0824	0.0903	0.3070
				0.0981, 0.1181, 0.1454, 0.3070		
			5	0.0334, 0.0368, 0.0527, 0.0670	0.0702	0.1407
				0.0734, 0.0949, 0.1246, 0.1407		

*PHI* pre-harvest interval, *STMR* supervised trials median residue, *HR* highest residue.

*The mean value of three parallel samples and all the final residues data arranged with the ascending order.

^#^Sum of BYI08330, B-enol, B-mono, B-keto and B-glu.

Currently, the MRL for thiacloprid in cowpeas has not yet been established in China and the MRL for spirotetramat in cowpeas is currently only a temporary limit value. This may be due to insufficient data on the dissipation and residue data of thiacloprid and spirotetramat in cowpeas in China. However, the results in this paper can provide more data for revising the application of thiacloprid and spirotetramat in cowpeas and establishing and confirming the MRL of thiacloprid and spirotetramat in cowpeas. At the same time, the residual data in this study can provide guidance for the proper and safe use of thiacloprid and spirotetramat and safety risk assessment.

### Dietary risk assessment

For the safe application of thiacloprid and spirotetramat, the risk quotient (RQ) was used to evaluate dietary risk assessment based on the terminal residues data in cowpeas. RQ < 100% indicates that the risk to humans is acceptable, whereas RQ value > 100% indicates that there is an adverse effect on human health^4,42^. Based on the JMPR reports^12,13^, the ADI of thiacloprid and spirotetramat are 0.01 mg kg^−1^ b.w. and 0.05 mg kg^−1^ b.w., respectively. The ARfD of thiacloprid and spirotetramat are 0.03 mg kg^−1^ b.w. and 1.0 mg kg^−1^ b.w., respectively. In Table 5, the different age individual vegetable intakes and body weights in China are listed according to the official summary report^43^. The STMRs and HRs values of thiacloprid and spirotetramat at PHI 3 days were listed in Table 4. The RQ_c_ and RQ_a_ values of thiacloprid in cowpeas for different Chinese consumers were 2.44–4.41% and 8.72–15.78%, respectively, and those of spirotetramat were 1.03–1.87% and 0.18–0.32%, respectively (Table 6). The highest risk crowd of dietary intake of thiacloprid and spirotetramat was for 2–7 years old children, and the lowest risk was 65 years old man. Interestingly, the chronic dietary exposure risk of thiacloprid in cowpeas was lower than the acute dietary exposure risk, while the chronic dietary exposure risk of spirotetramat in cowpeas was higher than the acute dietary exposure risk. And the risk of chronic and acute dietary intake of thiacloprid in cowpeas were all higher than spirotetramat. While both of the dietary risk levels were less than 100%. Therefore, if 22% thiacloprid and spirotetramat SC is applied on cowpeas at the recommended highest dosage and applying frequency, the potential health risk of thiacloprid and spirotetramat in cowpeas could be negligible to the health of different age consumers.

**Table 6:** Risk assessment of thiacloprid and spirotetramat in cowpeas for different groups of Chinese consumers.

Age	Sex	Body weight (kg)	Vegetable F_i_ (g day^−1^)	Thiacloprid		Spirotetramat	
				RQ_c_ (%)	RQ_a_ (%)	RQ_c_ (%)	RQ_a_ (%)
2–7	–	17.9	185.4	4.41	15.78	1.87	0.32
8–12	–	33.1	272.4	3.51	12.54	1.49	0.25
13–19	Male	56.4	328.1	2.48	8.86	1.05	0.18
	Female	50.0	336.5	2.87	10.25	1.22	0.21
20–50	Male	63.0	379.8	2.57	9.18	1.09	0.19
	Female	56.0	356.2	2.71	9.69	1.15	0.20
51–65	Male	65.0	390.3	2.56	9.151	1.08	0.184
	Female	58.0	352.9	2.59	9.27	1.10	0.19
> 65	Male	59.5	340.7	2.44	8.72	1.03	0.18
	Female	52.0	316.7	2.59	9.28	1.10	0.19

*RQ*_*c*_ risk quotient of the chronic dietary exposure, *RQ*_*a*_ risk quotient of the acute dietary exposure.

Based on the HR values and dietary risk assessment results of thiacloprid and spirotetramat, the MRL of thiacloprid for cowpeas can be tentatively set as 1.5 mg kg^−1^ according to the guidelines requirements for dietary risk assessment in China. While the temporary MRL of spirotetramat (5.0 mg kg^−1^) for cowpeas in China cannot fully guarantee the safety of cowpeas, and refer to MRL of spirotetramat in CAC (1.5 mg kg^−1^), EU (1.5 mg kg^−1^), Korea (1.5 mg kg^−1^), Australian (2.0 mg kg^−1^) and Japan (3.0 mg kg^−1^), a stricter maximum limit of spirotetramat residues in the cowpeas should be developed. The results from field trials and dietary risk assessment showed that thiacloprid and spirotetramat application on cowpeas in a manner of the good agricultural practices (GAP) is safe in China.

## Conclusion

In this study, a simple and easy residue analytical method was established for thiacloprid, spirotetramat and its four metabolites residue in cowpeas, and the validated method was utilized to investigate the degradation behavior and residue distribution of these pesticides in cowpeas under field conditions. The degradation half-lives of thiacloprid and spirotetramat ranged from 1.14–1.54 to 1.25–2.79 days, respectively, which showed that thiacloprid and spirotetramat degrades relatively quickly. The terminal residues of thiacloprid and spirotetramat were 0.0255–0.4570 mg kg^−1^ and 0.0314–0.3070 mg kg^−1^ after application of 22% thiacloprid and spirotetramat SC 2 times with at PHI 3 days. The RQ_c_ and RQ_a_ values of thiacloprid in cowpeas for different age consumers were 2.44–4.41% and 8.72–15.78%, respectively, and those of spirotetramat were 1.03–1.87% and 0.18–0.32%, respectively. These results indicated that thiacloprid and spirotetramat have a low dietary intake risk in cowpeas. According to the actual field application characteristics of thiacloprid and spirotetramat and their dietary intake risk assessment results, we have three recommendations: (1) When 22% SC of thiacloprid and spirotetramat is used to prevent and control pests on cowpeas, the maximum application time should only be twice at the highest recommended dose, and the PHIs should be 3 days; (2) the MRL of thiacloprid in the cowpeas can be tentatively set as 1.5 mg kg^−1^ for cowpeas, and a stricter maximum residues limit of spirotetramat in the cowpeas should be developed. (3) The 22% SC of thiacloprid and spirotetramat can be application on cowpeas, but the risk management measures should be implemented to ensure the safety of cowpea to consumers.

The results from our study are important reference for developing criteria for the safe use of thiacloprid and spirotetramat, developing maximum residue limits and ensuring the quality safety of agricultural products and consumers health.

## Supplementary Information

Supplementary Figure S1.
