# Supplementary figures and images for: Determination, residue analysis and risk assessment of thiacloprid and spirotetramat in cowpeas under field conditions
Source: Sci Rep. 2022 Mar 2;12:3470. doi: 10.1038/s41598-022-07119-1 (PMC8891356; doi:10.1038/s41598-022-07119-1)

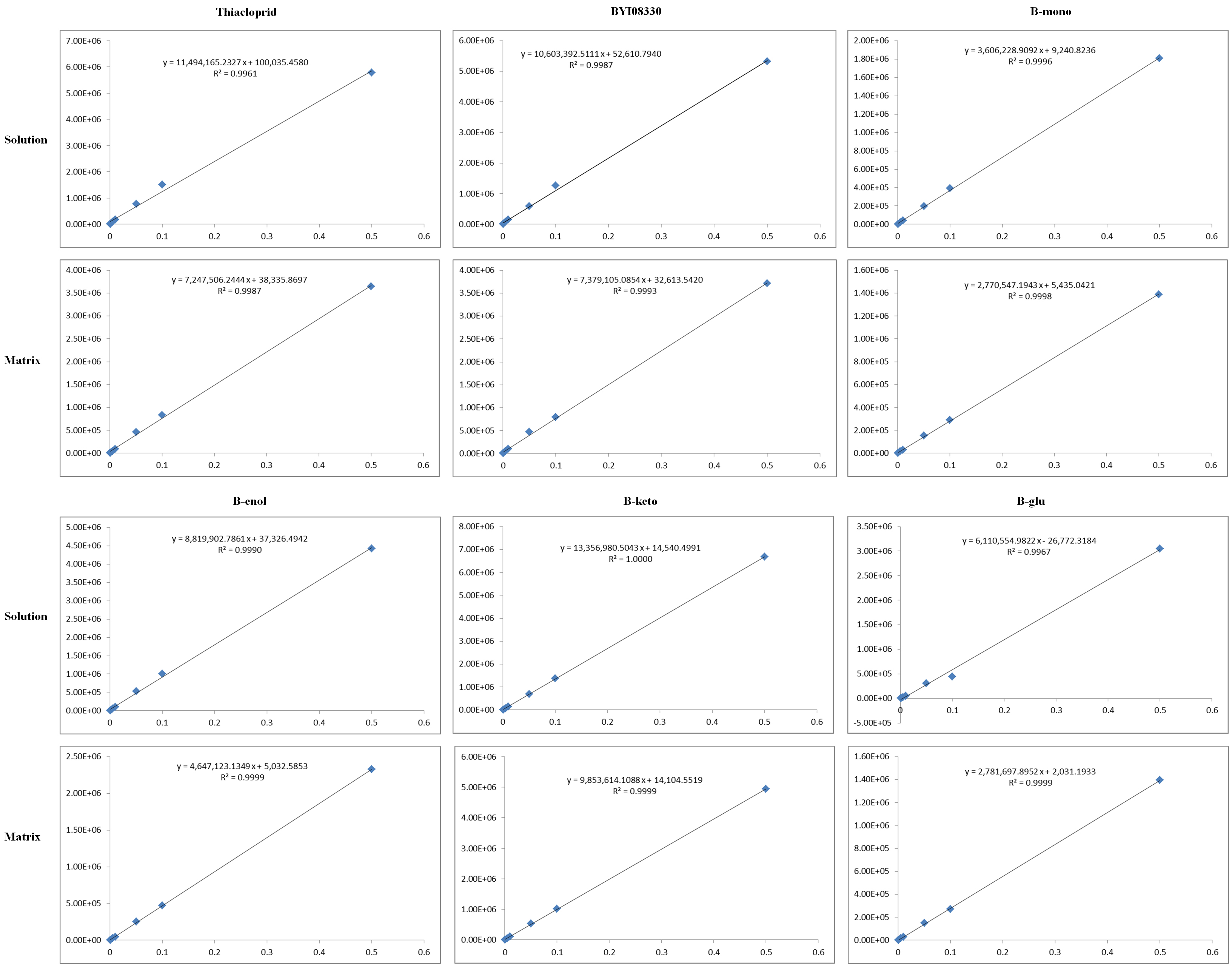

Supplement: Supplementary file 1 — Supplementary Figure S1. [file 41598_2022_7119_MOESM1_ESM.jpg]
